# Direct Production of Furfural in One-pot Fashion from Raw Biomass Using Brønsted Acidic Ionic Liquids

**DOI:** 10.1038/s41598-017-13946-4

**Published:** 2017-10-18

**Authors:** Babasaheb M. Matsagar, Shahriar A. Hossain, Tofazzal Islam, Hatem R. Alamri, Zeid A. Alothman, Yusuke Yamauchi, Paresh L. Dhepe, Kevin C.-W. Wu

**Affiliations:** 1 Catalysis & Inorganic Chemistry Division, CSIR-National Chemical Laboratory, Dr. Homi Bhabha Road, Pune, 411 008 India; 2 Academy of Scientific and Innovative Research (AcSIR), New Delhi, 110 025 India; 3 Department of Chemical Engineering, National Taiwan University, No. 1, Sec. 4, Roosevelt Road, Taipei, 10617 Taiwan; 4 International Center for Materials Nanoarchitectonics (MANA), National Institute for Materials Science (NIMS), 1-1 Namiki, Tsukuba, Ibaraki, 305-0044 Japan; 5 Australian Institute for Innovative Materials (AIIM), University of Wollongong, Squires Way, North Wollongong, NSW 2500 Australia; 6 Department of Biotechnology, Bangabandhu Sheikh Mujibur Rahman Agricultural University, Gazipur, 1706 Bangladesh; 7 Physics Department, Jamoum University College, Umm Al-Qura University, Makkah, 21955 Saudi Arabia; 8 Advanced Materials Research Chair, Chemistry Department, College of Science, King Saud University, Riyadh, 11451 Saudi Arabia; 9 School of Chemical Engineering & Australian Institute for Bioengineering and Nanotechnology (AIBN), The University of Queensland, Brisbane, QLD 4072 Australia

## Abstract

The conversion of raw biomass into C5-sugars and furfural was demonstrated with the one-pot method using Brønsted acidic ionic liquids (BAILs) without any mineral acids or metal halides. Various BAILs were synthesized and characterized using NMR, FT-IR, TGA, and CHNS microanalysis and were used as the catalyst for raw biomass conversion. The remarkably high yield (i.e. 88%) of C5 sugars from bagasse can be obtained using 1-methyl-3(3-sulfopropyl)-imidazolium hydrogen sulfate ([C_3_SO_3_HMIM][HSO_4_]) BAIL catalyst in a water medium. Similarly, the [C_3_SO_3_HMIM][HSO_4_] BAIL also converts the bagasse into furfural with very high yield (73%) in one-pot method using a water/toluene biphasic solvent system.

## Introduction

Lignocellulosic biomass, which comprises cellulose, hemicellulose, and lignin, is an excellent alternative to fossil feedstock for the synthesis of biochemical and biofuels. For example, furfural is one of the top 30 biomass-derived platform chemicals according to U.S. Department of Energy^1^. Typically furfural is mostly produced from xylose or xylan (hemicellulose) using the acid catalyst^2–4^. The acid hydrolysis of xylan into xylose and successive dehydration of xylose will produce furfural. Xylan is found in large quantity in lignocellulosic biomass such as bagasse, wheat straw, corn cob, rice husk, cotton stalk and jute. Therefore, if furfural production is carried out in a one-pot method using xylan-rich raw biomass, then it is possible to develop a cost-effective method for the synthesis of furfural.

Some reports have shown the conversion of raw biomass into C5 sugars (73% yield using HCl) and furfural by using mineral acid and solid acid as catalysts (e.g. 55% yield using H_2_SO_4_; 62% and 42% yields using Hβ and HMOR zeolites, respectively)^5–7^. However, the problem with these reported methods is their complicated reaction process (i.e. the reactions were carried out in more than one step). As it is understood that for the conversion of raw biomass into C5 sugars, and furfural, it is critical to pretreat the raw biomass for separating hemicellulose from lignocellulosic biomass and then breaking its rigid framework^8–10^. After that, the pretreated hemicellulose is then converted into C5 sugars and further into furfural^11^. Previously one-pot conversion of bagasse was reported using an acidic solid catalyst (i.e. HUSY with Si/Al = 15), but the yields of products were low (i.e. 20% (xylose + arabinose) and 55% furfural)^12^. In addition, it was also reported that the solid acidic catalyst was not stable under reaction conditions^12^. These previous studies indicated that the mineral acids and solid acids used for the conversion of raw biomass have several drawbacks: (1) they are not recyclable and (2) they make the reaction system more corrosive, which increases the capital cost^7,13^.

Combining ionic liquids (ILs) with mineral or solid acids is a resolution for efficient xylose-to-furfural conversion. For example, it was reported that the verity of catalysts (i.e. CrCl_3_, AlCl_3_, HCl CuCl, H_3_PO_4_, Amberlyst-15, LiCl, etc.)^8,14,15^ in a 1-butyl-3-methylimidazolium chloride ([BMIM][Cl]) IL system for converting xylose and xylan to furfural with a high yield (10–84% furfural yield from xylan). The use of acidic ILs was also reported for the chitosan conversion to HMF^16^. However, in these reactions, a large quantity of ILs was used (2 g IL for 38 mg of xylan) and thus the separation of products becomes very difficult, in addition to a huge cost of expensive ILs.

**Figure 1:**
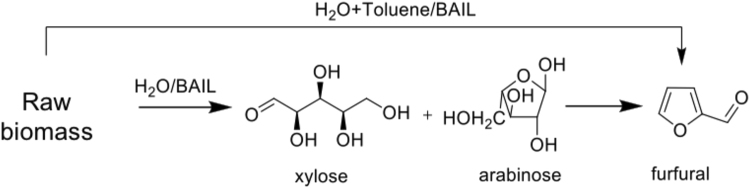
Conversion of raw biomass into C5 sugars and furfural in the one-pot method.

To overcome the drawbacks associated with the solid acid and mineral acid catalysts, many pioneering studies have utilized acidic ILs for the production of 5-hydroxymethylfurfural (HMF) and furfural from C6 and C5 sugar, respectively^17–20^. It is known that the world’s annual production of crops is very high^21^ (ESI; Table S1). After utilizing the edible part of all these crops, huge quantity of non-edible raw biomass is produced. Hence our aim is to use non-edible biomass for the production of C5 sugars followed by the C5 sugars-to-furfural conversion, as shown in Fig. 1. Here we present the use of a series of recyclable BAILs as the catalyst for the production of C5 sugars and furfural directly from raw biomass in a one-pot fashion. We believe our strategy would significantly enhance the efficacy of raw biomass conversion by using recyclable BAIL catalysts.

## Results and Discussion

### Catalyst evaluation: processing raw biomass into C5 sugars

**Figure 2:**
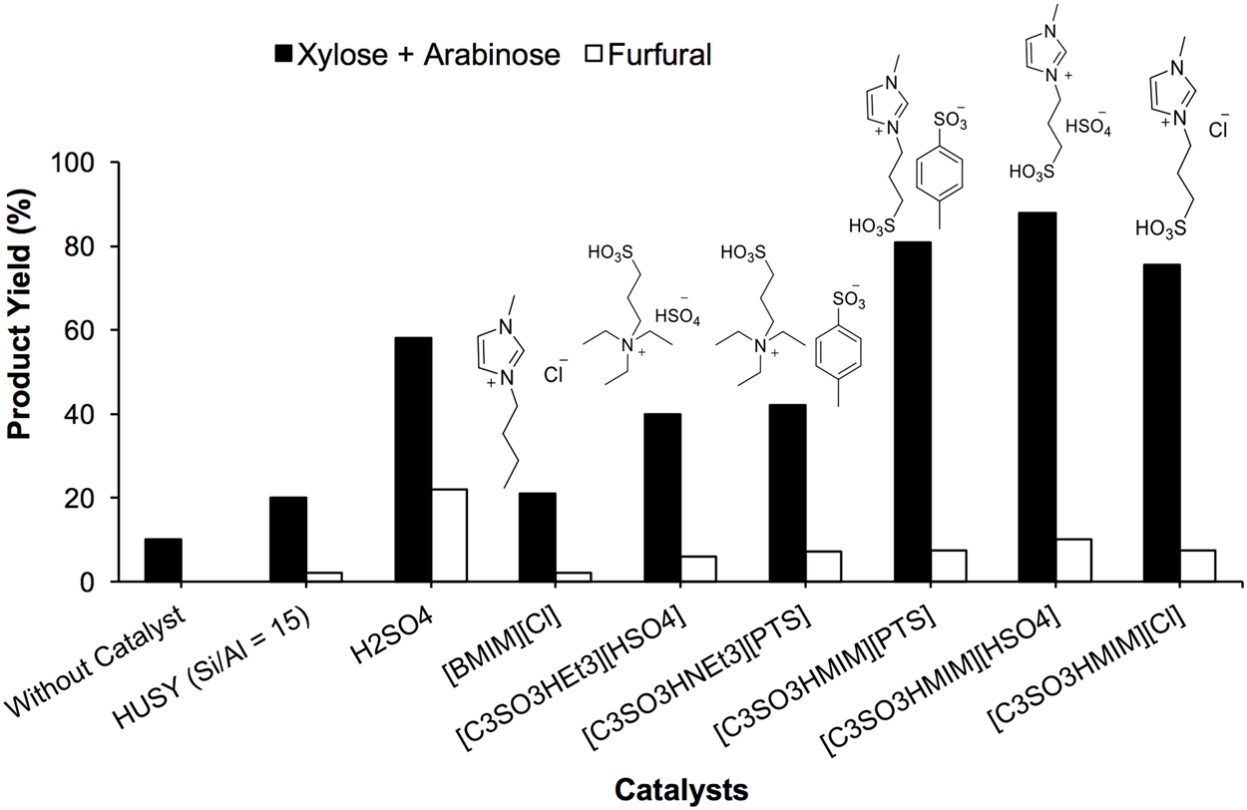
Catalyst evaluation study for processing of BG (I) into C5 sugars; Reaction condition: BG (I) (2 g), catalyst (0.24 g), H_2_O (60 mL), 160 °C, 1 h.

The catalytic performance of various BAIL-based catalysts for the conversion of the raw biomass (Bagasse (I)) to C5 sugars is summarized in Fig. 2. The reactions were performed in a water medium (60 mL) at 160 °C for 1 h. When 2 g of the Bagasse (I) was used the imidazolium-based BAILs such as [C_3_SO_3_HMIM][HSO_4_], [C_3_SO_3_HMIM][PTS] and [C_3_SO_3_HMIM][Cl] exhibited higher yields of 88%, 81% and 75% C5 sugar (xylose + arabinose), respectively, than those catalyzed by mineral acid like H_2_SO_4_ (58% C5 sugar yield) and solid acidic catalyst like HUSY (Si/Al = 15) (20% C5 sugar yield)^19^. In previous study HCl catalyst was used for the conversion of isolated hemicellulose into C5 sugars. The result showed lower C5 sugar yield (39%) compared to [C_3_SO_3_HMIM][HSO_4_] BAIL (87% C5 sugar yield) catalyst. Hence, in the present work, HCl catalyst was not used for the conversion of raw biomass^19^. For comparison, the non-catalytic reaction showed only 10% yield of C5 sugars under similar reaction conditions, indicating that the BAIL catalysts indeed promote the conversion of Bagasse to C5 sugars. The reaction carried out using quaternary ammonium-based BAILs (e.g. [C_3_SO_3_HNEt_3_][HSO_4_] and [C_3_SO_3_HNEt_3_][PTS]) showed lower C5 sugar yields (i.e. 40% and 42%, respectively). This indicates that imidazolium-containing BAILs are more efficient catalysts. We suggest that the reason is that of the presence of imidazolium cation in the BAIL can have ion-dipole type of interaction with polysaccharides, thus an easier availability of H^+^ for the hydrolysis of pentosan in raw biomass can be achieved. In contrast, in the case of quaternary ammonium-based BAILs, the structure of cations is not planar so the interaction between BAILs and polysaccharides was not so achievable and therefore showed lower yields of C5 sugars. Along with ion-dipole interaction, acid strength is also one of the factors which decide the activity. The difference in activity among BAILs as shown in Fig. 2 can be seen because of the dissimilar acid strength of the acidic ILs. The [C_3_SO_3_HMIM][HSO_4_] BAIL has highest acid strength ([C_3_SO_3_HMIM][HSO_4_] *Ho* = 2.08, [C_3_SO_3_HMIM][PTS] *Ho* = 2.33, [C_3_SO_3_HMIM][Cl] *Ho* = 2.47) and hence showed better activity compared to all other acidic ILs^19^. In addition, to understand the contribution of Brønsted acidity in the processing of crop waste (BG (I)) into C5 sugars, we also tested a non-Brønsted acid ionic liquid (i.e. [BMIM][Cl]). The [BMIM][Cl] exhibited only 21% yield, which indicated that the higher yield of C5 sugars was resulted from the Brønsted acidity of BAIL. Apart from C5 sugars formation, C6 sugar (glucose and fructose, 3–4%), furfural (6–10%) and HMF (2–3%) were also found to be present in the final product. However, no any aromatic products derived from lignin were observed, suggesting a selective conversion of the pentosan part from bagasse to C5 sugars.

### Effect of the type of raw biomass

**Figure 3:**
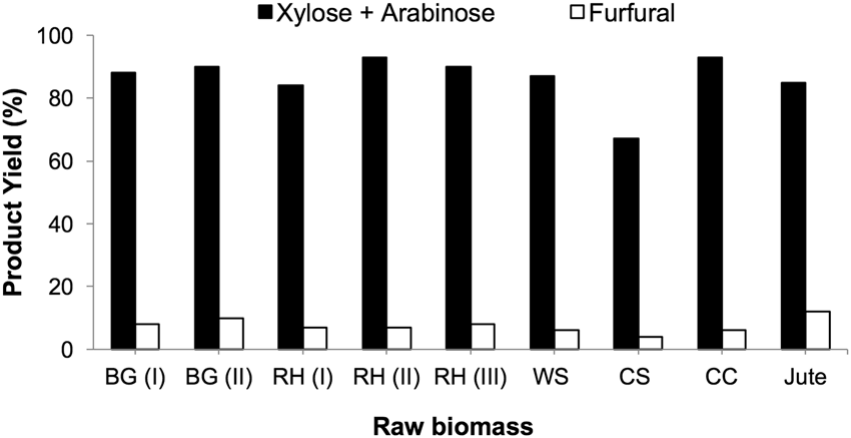
Processing of raw biomass into C5 sugars; Reaction conditions: raw biomass (2 g), H_2_O (60 mL), [C_3_SO_3_HMIM][HSO_4_] (0.24 g), 160 °C, 1 h.

After obtaining an exceptionally high yield of C5 sugars (88%) from BG (I) crop waste using the [C_3_SO_3_HMIM][HSO_4_] BAIL, we applied this BAIL to a wide range of crop wastes including bagasse (BG), rice husk (RH), wheat straw (WS), cotton stalk (CS), corn cob (CC) and jute. All reactions were carried out at 160 °C for 1 h in a water medium (60 mL). As shown in Fig. 3, the results showed that almost all types of raw biomass could be efficiently converted into C5 sugars. The yields of C5 sugars were calculated based on the amount of pentosan in raw biomass (Table S3, ESI). All the experiments were repeated 3 times, and the error in the yields of C5 sugars was ±3%. Without the addition of any mineral acid or metal halide, the method used for converting raw biomass such as bagasse, rice husk, wheat straw, cotton stalk, corn cob, and jute into C5 sugars is efficient with an average yield above 80%. Furthermore, we have confirmed that only hemicellulose in crop waste was converted into C5 sugars while lignin present in crop waste remained intact. This developed method can be further scaled up for producing C5 sugars with a very high yield from pentosan-rich raw biomass.

### Processing of raw biomass into furfural

**Figure 4:**
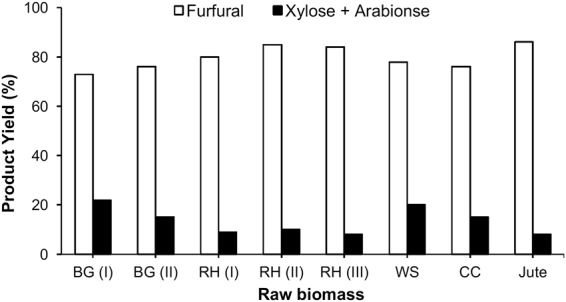
Processing of crop wastes into furfural; Reaction condition: crop waste (0.6 g), [C_3_SO_3_HMIM][HSO_4_] (0.12 g), H_2_O and Toluene mixture (1:5 *v/v*; 60 mL), 170 °C, 3 h.

After selectively converting raw biomass into C5 sugars using [C_3_SO_3_HMIM][HSO_4_] BAIL successfully, we further exploited [C_3_SO_3_HMIM][HSO_4_] BAIL for the conversion of hemicellulose in raw biomass into furfural. A variety of raw biomass (0.6 g) including bagasse (BG), rice husk (RH), wheat straw (WS), cotton stalk (CS), corn cob (CC) and jute were used as reactants, and the conversion was performed at 170 °C for 3 h in a water/toluene biphasic system with the presence of the [C_3_SO_3_HMIM][HSO_4_] BAIL (0.12 g). As shown in Fig. 4, the results clearly indicated that almost all types of raw biomass were successfully converted into furfural with very high yields (73–88%). Rice husk collected from agricultural field of different places of India [RH (I, II, and III)] showed >80% furfural yield while the bagasse collected from agricultural field of different places of India [BG (I, II)] showed 73% and 76% furfural yield along with 22% and 15% C5 sugar yield, respectively. Jute obtained from Bangladesh showed 86% furfural yield. Finally, the wheat straw and corn cob derived biomass showed 78% and 76% furfural yield along with 20% and 15% C5 sugar yield, respectively. Our [C_3_SO_3_HMIM][HSO_4_] BAIL catalyst exhibited its superior ability to convert various raw biomass to furfural in one-step reaction.

In the reaction of the selective conversion of hemicellulose in BG (I) into furfural reaction, over 99% conversion of pentosan was achieved, and 73% yield of furfural along with 22% yield of C5 sugars was obtained. In contrast, cellulose and lignin in BG (I) were intact because no products derived from cellulose and lignin were found in a substantial amount. In these reactions, the mass of solid recovered after the reaction was calculated the result suggests that 98% mass balance was achieved. For example, 0.6 g BG (I) (which contains 0.145 g pentosane and 0.455 g cellulose and lignin, respectively) was used for the reaction. After reaction, 0.451 g of solid was recovered. This result showed that only pentosan was consumed and all the other parts such as cellulose and lignin remained intact.

### Pretreatment of cellulose

The pentosan presenting in raw biomass was completely converted into C5 sugars and furfural using [C_3_SO_3_HMIM][HSO_4_] BAILs. Along with C5 sugars and furfural, 4–6% yield of C6 sugars (glucose and fructose) was also detected when the reaction temperature and time increased to 170 °C and 3 h, respectively. This result suggests that cellulose can be converted into sugars when the temperature and time of the reaction are increased. To understand how much the cellulose exactly converted into products from raw biomass, the reaction of pure microcrystalline cellulose was performed under similar reaction conditions (170 °C, 3 h) using the [C_3_SO_3_HMIM][HSO_4_] BAIL catalyst. The result showed 10% conversion of cellulose and 7% yield of the products including glucose, fructose and HMF. This implies that very little cellulose was converted in the presence of BAIL catalyst.

**Figure 5:**
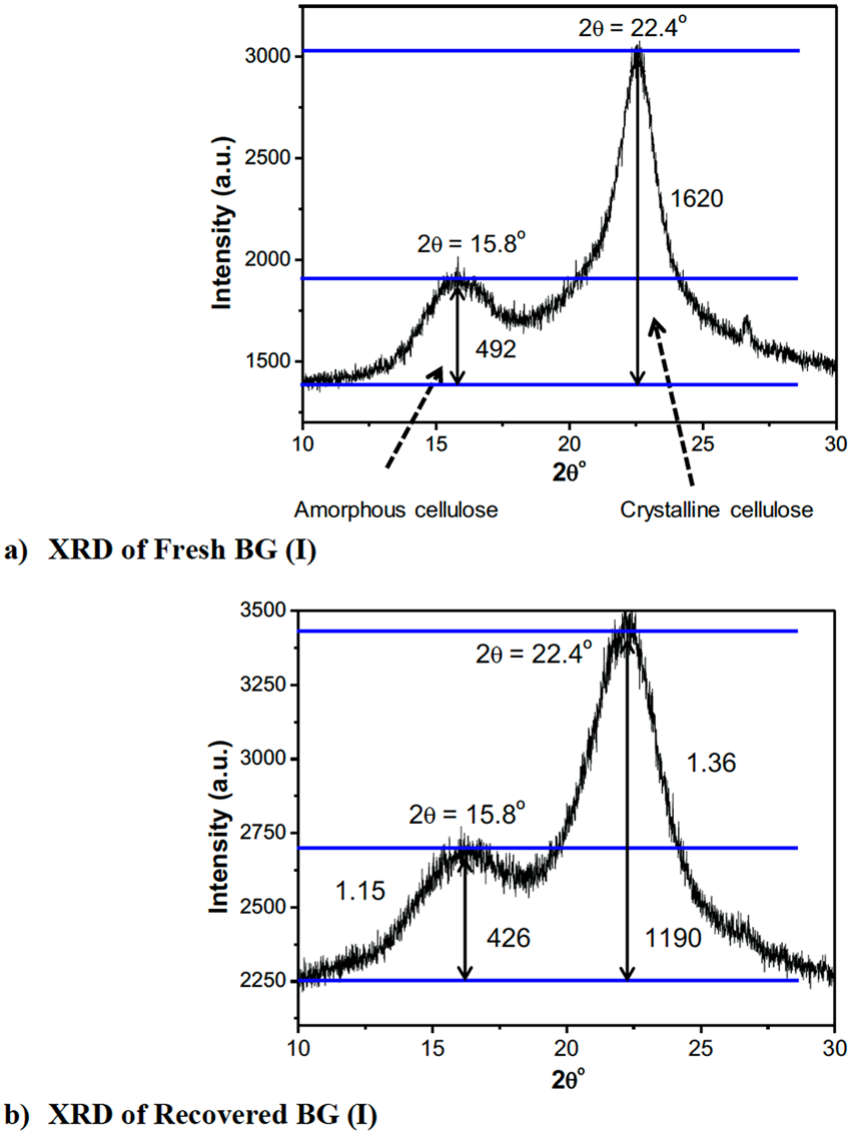
XRD patterns of (**a**) fresh and (**b**) recovered BG (I). Reaction condition: BG (I) (2 g), [C_3_SO_3_HMIM][HSO_4_] (0.24 g), H_2_O (60 mL), 170 °C, 3 h.

To understand the structural change of cellulose in BG (I) upon catalytic reaction, the crystallinity of cellulose in BG (I) was examined before and after the reaction. The process used for the recovery of solid from reaction for XRD analysis is explained as follows: 2.0 g of BG (I) was used for the reaction, and after the reaction solid was separated using filtration. The solid recovered from the reaction mixture was then dried at 60 °C for 16 h. Next, the solid was vacuum dried at 100 °C for 12 h before XRD analysis. As shown in Fig. 5(a), there are peaks for fresh BG (I) sample in the XRD, showing the amorphous and crystalline cellulose peaks at 15.8° and 22.4°, respectively. After the catalytic conversion of BG (I) with the [C_3_SO_3_HMIM][HSO_4_] BAIL at 170 °C for 3 h, the residual sample exhibited same XRD peaks for amorphous and crystalline cellulose (i.e. same peak position but lower peak intensity), as shown in Fig. 5(b). The decrease of the XRD peak intensity indicated that the crystallinity of cellulose decreased after the catalytic reaction in the presence of the [C_3_SO_3_HMIM][HSO_4_] BAIL catalyst.

Many studies have reported that the imidazolium-based ILs are useful for the pretreatment of crystalline cellulose, which decreases the crystallinity of cellulose^22–24^. This is because that ILs can facilitate the solubility of cellulose and increase the hydrolysis of cellulose in the presence of an acid catalyst. However, generally a huge quantity of ILs is required for such pretreatment (e.g. 2 g IL for 0.1 g cellulose). In this study, it is showed that a small amount of BAIL such as [C_3_SO_3_HMIM][HSO_4_] can also decrease the crystallinity of cellulose in raw biomass, although there was very little cellulose conversion.

### Recycle study for [C_3_SO_3_HMIM][HSO_4_] BAIL

Recycling the catalyst is always an important issue for a novel catalyst, so we performed the recycling experiments of the [C_3_SO_3_HMIM][HSO_4_] BAIL catalyst in this study. After the first reaction, the solid present in the reaction mixture was separated using filtration, and then the toluene and water layers were separated using a separating funnel. Because the furfural is soluble in toluene and the [C_3_SO_3_HMIM][HSO_4_] BAIL is not soluble in toluene, a small amount of furfural soluble in aqueous layer was extracted using fresh toluene solvent (3 times extraction). The residual aqueous layer contained soluble IL and a few sugars. This water-containing IL was then used for the next reaction (Fig. S6, ESI). Subsequent reactions were carried out by decreasing the substrate and solvent quantity (to keep substrate/catalyst ratio and the substrate/solvent ratio constant). As the quantity of aqueous layer decreased in every subsequent reaction, accordingly the substrate and solvent quantity were changed. The C5 sugars presenting in aqueous layer was considered for calculating the final yield of furfural.

**Figure 6:**
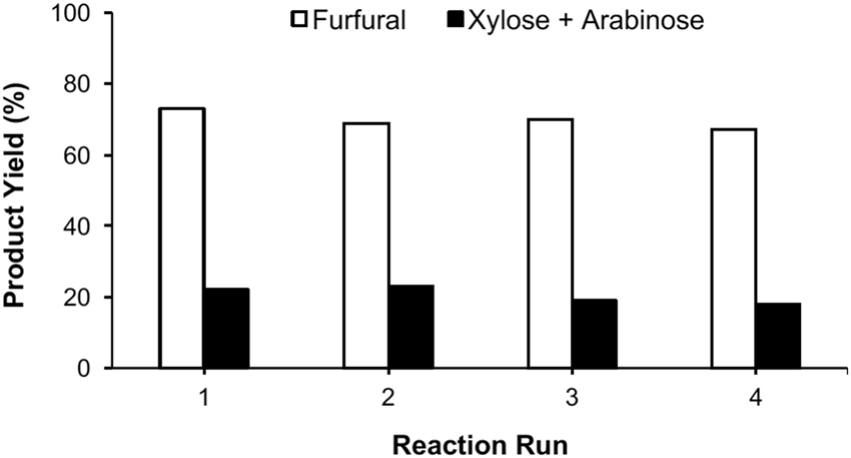
Recycle study for [C_3_SO_3_HMIM][HSO_4_] BAIL used for processing of BG (I) into furfural; Reaction condition: BG (I)/[C_3_SO_3_HMIM][HSO_4_] (4.8 wt%), H_2_O and Toluene mixture (1:5 v/v) 170 °C, 3 h.

The reactions were carried out at 170 °C for 3 h in the water/toluene biphasic solvent system, and the substrate to catalyst ratio was kept constant for all reactions. As shown in Fig. 6, the recycle test showed an almost similar yield of furfural for the first 4 runs. The yield of furfural was 73% in the very beginning. In the first recycle run 69% furfural yield was obtained, and in the second and third recycle runs 70% and 67% furfural yields were obtained, respectively. In addition, 18–22% C5 sugar yields were obtained for all reactions. The results of recycle test indeed indicated that the recyclability and stability of the presenting [C_3_SO_3_HMIM][HSO_4_] BAIL.

### Study of metal exchange

As the BAILs used in the present study are acidic in nature because of the presence of the –SO_3_H group, there may be a possibility that H^+^ of BAILs can be exchanged with the metal ions which are presenting in the raw biomass. The loss of H^+^ can therefore destroy the acid sites of BAIL. For this reason, we attempt to understand whether there is an exchange of H^+^ of BAILs with metal ions in the reaction mixture (i.e. processed BG (I) into furfural using [C_3_SO_3_HMIM][HSO_4_] BAIL) using ICP-OES technique. As presented in Table 1, very little quantity of metal nutrients is presenting in BG (I) (i.e. 0.06 mmol for 0.6 g of BG (I)). ICP-OES results showed that K (0.01 mmol/g) and Ca (0.01 mmol/g) were found in reaction solution but their quantity is again very less [0.012 mmol K and Ca for 0.6 g BG (I)] Other than these two metal ions, there was no other metal ion detected in the reaction solution. This result indicated that there was a very low possibility for a considerable exchange of metals with the H^+^ of BAILs. Thus, BAILs can be recycled and retained good catalytic ability for the selective conversion of raw biomass to furfural.

**Table 1:** ICP-OES results for metal exchange study.

Nutrients (mmol/g)	Fresh BG (I)	Reaction mixture	Recovered solid from reaction mixture
Na	0	0	0
K	0.03	0.01	0.02
Ca	0.03	0.01	0.02
Mg	0.02	0	0.02
Al	0.01	0	0.01
P	0.01	0	0.01

ICP-OES analysis; calculations were done based on 1 g of BG (I).

## Conclusion

In this study, a successful and efficient method for the conversion of various kinds of raw biomass to C5 sugars and then to furfural in one-pot reaction using a series of BAILs was demonstrated. The [C_3_SO_3_HMIM][HSO_4_] BAIL showed the best results compared to all other ILs because of its acidic nature and planar structure of imidazolium cation. In the processing of BG (I) into C5 sugars using the [C_3_SO_3_HMIM][HSO_4_] BAIL catalyst, the maximum yield of C5 sugar was 88% under the conditions of 160 °C within 1 h. This value is higher than those catalyzed by conventional solid heterogeneous catalyst HUSY (Si/Al = 15) and homogeneous catalyst H_2_SO_4_ (i.e. 20% and 58%, respectively). Moreover, it is also demonstrated that the [C_3_SO_3_HMIM][HSO_4_] BAIL catalyst can be applied to various kinds of raw biomass including bagasse, rice husk, wheat straw, cotton stalk, corn cob, and jute with an exceptionally high yield of C5 sugars (67–93%) and furfural (76–86%). In present system, no mineral acid or metal halides were used. And the [C_3_SO_3_HMIM][HSO_4_] BAIL catalyst can be successfully recycled, indicating current method is more environmentally friendly and efficient that would be useful for other acid-assisted catalytic reactions.

## Experimental

### Materials and Method

For the raw biomass conversion, various types of raw biomass including rice husk, wheat straw, bagasse, cotton stalk, corn cob, and jute that were previously collected from agricultural fields located in different parts of India and Bangladesh are used. Before the experiments, the raw biomass was shattered using a mixer grinder and sieved to obtain ca. 2 mm size particles. Next, the raw biomass was washed (for removing residual sugars) with water at room temperature and dried in an oven at 60 °C for 16 h prior to being vacuum dried at 80 °C for 16 h. Afterward, these crop wastes were used in the reaction.

The materials and detailed synthesis procedure of BAIL are described in Section 2 & 3, ESI (Table S2 and Fig. S1). The synthesized BAILs were characterized with NMR (^1^H, ^13^C), FTIR, TGA, CHNS microanalysis and other analysis methods. for confirming the structure and stability of the BAILs^20^.

All the catalytic reactions were carried out at high pressure and high-temperature batch mode autoclave (Parr autoclave; 300 mL capacity) equipped with temperature manager unit. In a normal reaction, for the processing of raw biomass into C5 sugars, 2 g of biomass was charged in the reactor and then 60 mL of water along with 0.24 g catalyst (BAIL) was added to the reactor. For the processing of raw biomass into furfural in the one-pot method, 0.6 g of raw biomass was charged in the reactor and then the water and toluene biphasic solvent was added (60 mL; 1:5 *v/v*) along with 0.12 g catalyst (BAIL). Reactions were performed at desired reaction temperature (160 °C and 170 °C) for definite reaction time (1 h and 3 h) under mechanical stirring (800 rpm).

### Analysis

The analysis of samples was performed using HPLC instrument (Agilent) equipped with a Rezex RPM-Monosaccharide Pb^2+^ column (300 × 7.8 mm; particle size 8 μm) at 80 °C. Millipore water was used as an eluent with a flow rate of 0.6 mL/min. The refractive index detector (RID) with a cell temperature of 40 °C was used to detect the products. Before analysis, the samples were filtered through 0.22 μm syringe filter, which was then injected for the analysis. The calibration curve was plotted with the standard compounds for calculating the yield of products. The Gas chromatography (GC) equipped with HP-5 column was used for the analysis of the organic layer of the reaction mixture. The Flame Ionization Detector with the temperature 280 °C was used for the examination of verity of products.

### Calculations

The yield of sugars obtained by selective conversion of hemicellulose from crop waste was calculated based on the concentration of pentosan presenting in the raw biomass. Technical Association of the Pulp and Paper Industry (TAPPI) method was used for deciding the pentosan concentration in all raw biomass. The calculations of C5 sugar yield and furfural yield are shown in the Section 4 of ESI.

### Compositional analysis of raw biomass

TAPPI method was used to understand the composition of raw biomass (holocellulose, pentosan, lignin, ash, etc.) (more details are given in ESI Section 5). Various metal nutrients are used for the growth of plants, so there is a possibility of the presence of various metal nutrients in the non-edible biomass. The metal nutrients presenting in raw biomass were determined by ICP-OES analysis (Table S4, ESI)

## Electronic supplementary material

Supporting Information
