# Supplementary material for: Direct Production of Furfural in One-pot Fashion from Raw Biomass Using Brønsted Acidic Ionic Liquids
Source: Sci Rep. 2017 Oct 18;7:13508. doi: 10.1038/s41598-017-13946-4 (PMC5647444; doi:10.1038/s41598-017-13946-4)
Supplement: Supplementary file 1 — Supporting Information [file 41598_2017_13946_MOESM1_ESM.pdf]

Supporting Information

Direct Production of Furfural in One-pot Fashion from Raw Biomass Using Brønsted Acidic Ionic Liquids

Babasaheb M. Matsagar,[abc] Md. Shahriar A. Hossain,[de] Md. Tofazzal Islam,[f] Hatem R. Alamri,[g] Zeid A. Alothman,[h] Yusuke Yamauchi,[de] Paresh L. Dhepe,[ab]* and Kevin C.-W. Wu[c]*

a *Catalysis & Inorganic Chemistry Division, CSIR-National Chemical Laboratory, Dr. Homi Bhabha Road, Pune 411 008, India*

b *Academy of Scientific and Innovative Research (AcSIR), New Delhi 110 025, India*

c *Department of Chemical Engineering, National Taiwan University, No. 1, Sec. 4, Roosevelt Road, Taipei 10617, Taiwan*

d *International Center for Materials Nanoarchitectonics (MANA), National Institute for Materials Science (NIMS), 1-1 Namiki, Tsukuba, Ibaraki 305-0044, Japan*

e *Australian Institute for Innovative Materials (AIIM), University of Wollongong, Squires Way, North Wollongong, NSW 2500, Australia*

f *Department of Biotechnology, Bangabandhu Sheikh Mujibur Rahman Agricultural University, Gazipur 1706, Bangladesh*

g *Physics Department, Jamoum University College, Umm Al-Qura University, Makkah 21955, Saudi Arabia.*

h *Advanced Materials Research Chair, Chemistry Department, College of Science, King Saud University, Riyadh 11451, Saudi Arabia.*

E-mail address: pl.dhepe@ncl.res.in *and* kevinwu@ntu.edu.tw

1. Production of crops

Table S1. Production of crops (India and world scenario).

Production of crops in 2014 (MT)						
**Region/ country**	**Sugar cane**	**Rice, (paddy)**	**Wheat**	**Jute**	**Maize**	**Cotton lint**
**India**	$3.52x10^8$	$1.57x10^8$	$9.58x10^7$	$1.96x10^6$	$2.36x10^7$	$6.18x10^6$
**World**	$1.88x10^9$	$7.41x10^8$	$7.29x10^8$	$3.39x10^6$	$1.03x10^9$	$2.61x10^7$

2. Materials

For the synthesis of various ILs the precursors were procured from different sources. The details are given in Table S2.

Table S2. Summary of materials used for the synthesis of ILs.

**Chemical Name**	**CAS No.**	**Molecular Formula**	**Purity (%)**	**Supplier**
1-methylimidazole	616-47-7	$C_4H_6N_2$	99	Across Organics, USA
1-chlorobutane	109-69-3	C_4H_9Cl	99	Sigma-Aldrich, USA
1,3-propane sultone	1120-71-4	$C_3H_6O_3S$	99	Alfa Aesar, India
1-methylbenzimidazole	1632-83-3	$C_8H_8N_2$	99	Alfa Aesar, India
triethylamine	121-44-8	$C_6H_{15}N$	98	Alfa Aesar, India
p-toluenesulfonic acid monohydrate	6192-52-5	$C_7H_{10}O_4S$	98	Sigma-Aldrich, USA

All the above chemicals were used as received. The solid acid catalyst (Zeolites) HUSY (Si/Al=15) was procured from Zeolyst International (USA). Prior to use, HUSY (Si/Al=15) was calcined at 550 °C (temperature ramping 2 °C/min) for 16 h in an air flow (20 mL/min).

3. Synthesis of Catalyst

The typical method used for the synthesis of BAILs is shown in Scheme S1.

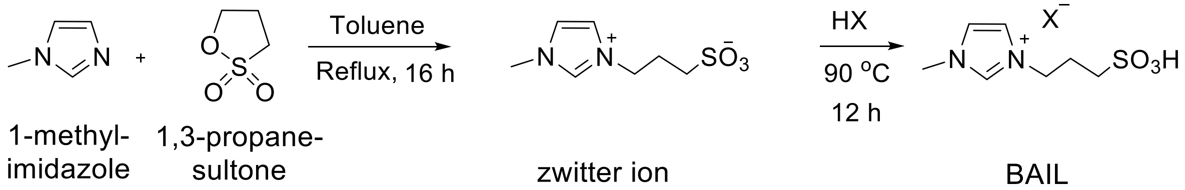

Fig. S1. Synthesis method of imidazolium based BAILs.

HX: HCl, H_2SO_4, PTSA; X: Cl^-, HSO_4^-, PTS.

4. Calculations

Calculation of C5 sugar (xylose+arabinose) and furfural yields for the selective conversion of hemicellulose from crop wastes into C5 sugars is done as follows,

$$\textbf{Xylose + Arabinose yield}\ (\%) = \frac{\text{moles of xylose + arabinose (HPLC)}}{\text{theoretical moles of xylose + arabinose}} \text{x } 100$$

$$\textbf{Furfural yield}\ (\%) = \frac{\text{moles of furfural (HPLC)}}{\text{theoretical moles of furfural}} \text{x } 100$$

Calculation of furfural yield for the reaction of the conversion of hemicellulose from crop wastes into furfural.

$$\textbf{Furfural yield}\ (\%) = \frac{\text{moles of furfural (HPLC + GC)}}{\text{theoretical moles of furfural}} \text{x } 100$$

5. Compositional analysis of raw biomass

5.1. Determination of ash and moisture content

Oven dried (O.D.) sample (100 °C for 12 h under vacuum) was taken for the analysis of ash content. O.D. sample (5 g) was taken in silica crucible (W1). The crucible was placed in a muffle furnace at 650 °C for 4 h in the presence of air. Afterwards the sample was cooled to 70 °C and then placed in a desiccator containing indicating-grade anhydrous alumina. After cooling the sample to room temperature, the weight of ash content with crucible was determined (W2).

$$\textbf{Ash content}\ (\%) = \frac{\text{W1} - \text{W2}}{\text{O. D. weight of the sample}} \text{x } 100$$

For the determination of moisture present in crop wastes, the crop wastes were dried in an oven at 100 °C for 12 h under vacuum. The amount of moisture was calculated using the difference in the weight of the sample before and after drying.

$$\textbf{Moisture content}\ (\%) = \frac{\text{Initial weight} - \text{Oven dried weight}}{\text{Initial weight}} \text{x } 100$$

5.2. Determination of pentosan in crop waste

Pentosan is a polysaccharide yielding only pentoses (C5 sugars) on hydrolysis. The distillation apparatus used for the determination of pentosan is shown in Fig. S1. HCl (13.5%) reagent was used for the determination of pentosan.

The stepwise procedure used for pentosan determination is shown in scheme S2.

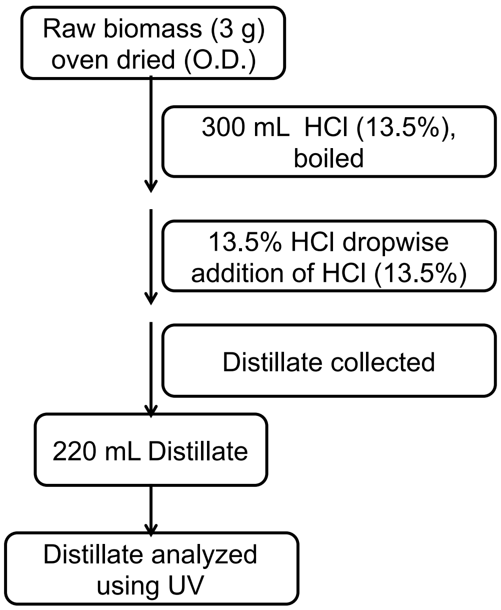

Fig. S2 Determination of pentosan

The absorbance of the distillate at 280 nm was measured using UV-spectrophotometer and the pentosan % was calculated using the below formula.

$$\textbf{Pentosan}\ (\%) = \frac{\text{Absrobance at 280 nm x Dilution factor x 1.563 x 0.5 x 100}}{\text{151 x O. D. weight of sample}}$$

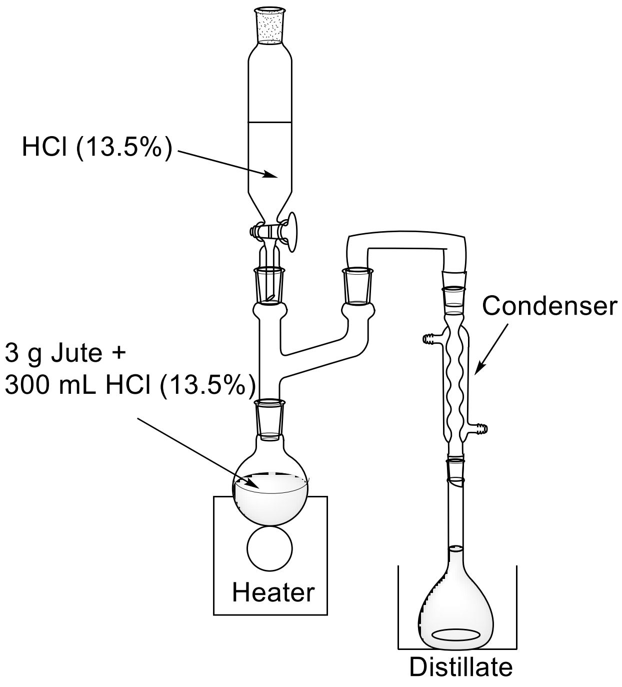

Fig. S3 Distillation apparatus used for pentosan determination

5.3. Determination of holocellulose

The holocellulose is nothing but the total carbohydrate content of the raw biomass material. The holocellulose comprises cellulose and hemicellulose part of lignocellulosic biomass. The holocellulose content was calculated using delignification method in which sodium chlorite ($NaClO_2$) and acetic acid (CH_3COOH) were used as the reagents.

5.3.1. Delignification of lignocellulosic biomass

For delignification, O.D. (3 g) sample was taken in 250 mL conical flask. 150 mL distilled water was added to it along with 1.5 g of sodium chlorite ($NaClO_2$) and 0.5 mL of acetic acid (CH_3COOH). Then the flask was placed in the water bath and was heated at 70 °C. The conical flask was covered with small flask in an inverted orientation. The reaction system was then heated for 1 h. After cooling, the supernatant was transferred to a tared crucible. The lignin is soluble in solution and lignin-free (holocellulose) pulp can be obtained after filtration. Nevertheless, some lignin was still present in holocellulose after this treatment. Hence, the treatment with sodium chlorite and acetic acid was repeated at least three times for complete delignification. Then the mixture in conical flask was filtered through tared crucible, and the obtained residue was then washed with acetone. Further, the residue was dried in oven at 100 °C for 2 h and the weight of filtering crucible with contents was recorded.

The difference in weight of O.D. crop waste and holocellulose pulp will give the amount of lignin. The holocellulose obtained in this treatment contains ash, therefore, for the determination of the exact amount of holocellulose, the weight of ash was subtracted form holocellulose.

5.4. Determination of acid-insoluble lignin in pulp

The lignin obtained in this method was called as uncorrected lignin. The uncorrected lignin was transferred to quartz boat and then it was heated at 650 °C for 4 h in the presence of air for ash correction.

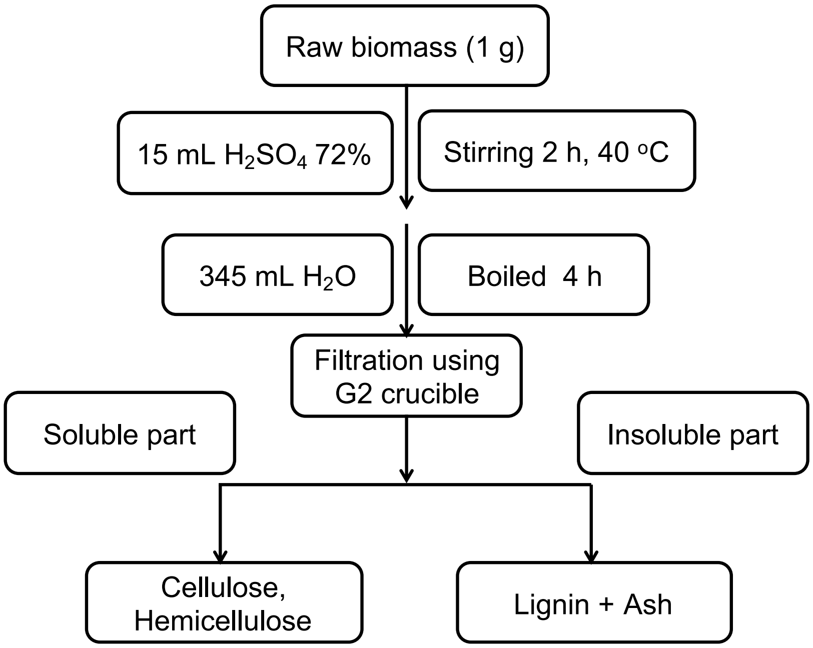

Fig. S4 Determination of lignin

$$\boldsymbol{wt}\ \textbf{of uncorrected lignin}\ (\mathrm{g}) = [wt\ \text{of (crucible + solid)} - wt\ \text{of empty crucible}]$$

$$\textbf{Ash corrected lignin}\ (\%) = \frac{[wt\ \text{of uncorrected lignin} - wt\ \text{of ash}]}{\text{O. D. weight of sample}}\ \mathrm{x}\ 100$$

5.5. Determination of α-cellulose, β-cellulose and γ-cellulose

Generally, the α-cellulose indicates undegraded, higher-molecular weight cellulose content in pulp; the β-cellulose indicates that of degraded cellulose, and the γ-cellulose consists mainly hemicellulose. The definition of α-cellulose is the pulp fraction resistant to 17.5% NaOH and 9.45% solution under reaction condition. β-cellulose is the soluble fraction which is re-precipitated on acidification of the solution, and lastly γ–cellulose is the fraction remaining in the solution.

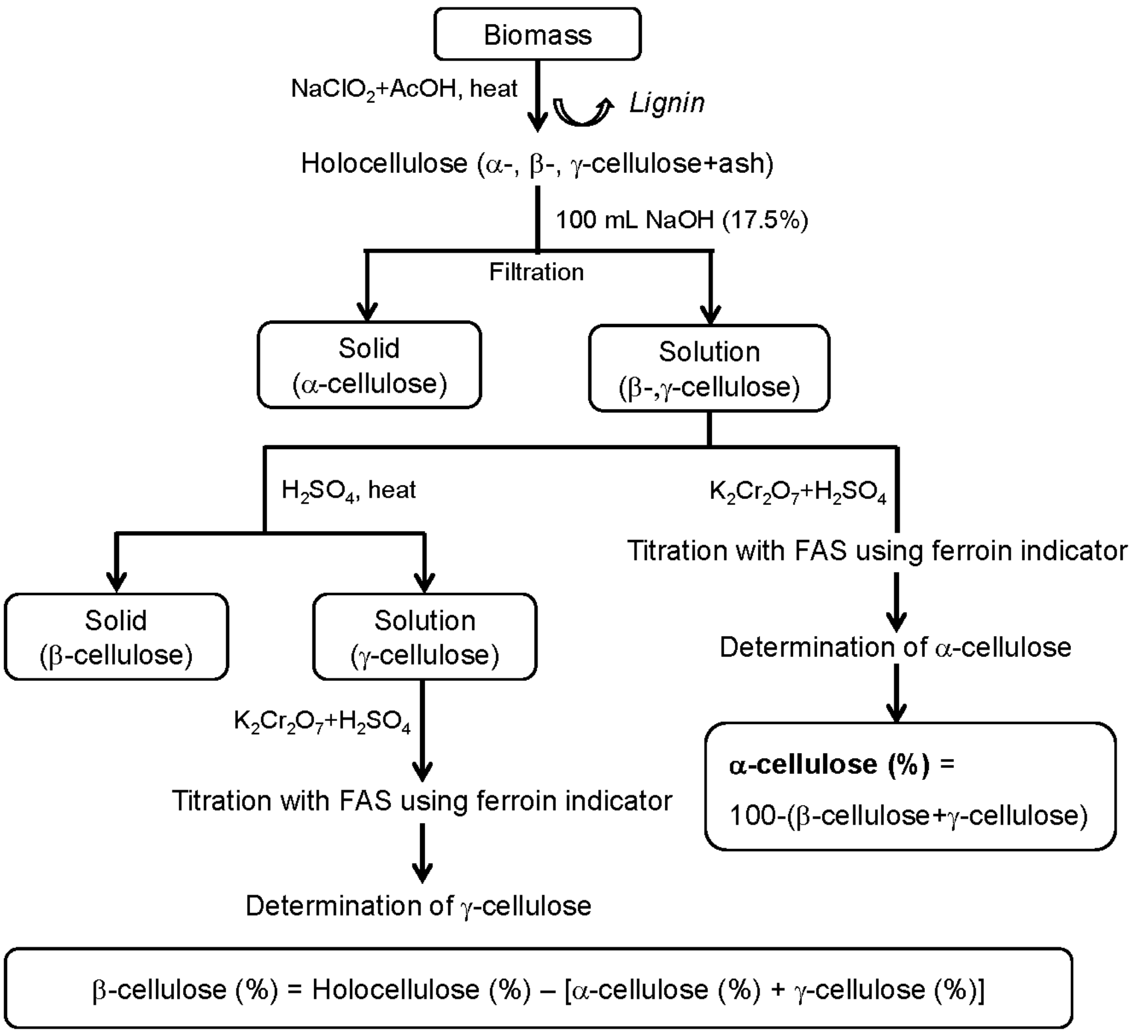

Fig. S5. Schematic for the determination of α-. β- and γ-cellulose.

Calculations:

Calculation of α-cellulose content:

α-cellulose (%) = $100-[(6.85\ (V_2-V_1) \times N \times 20)/A \times W]$

where:

V1 = titration of the pulp filtrate, mL

V2 = blank titration, mL

N = exact normality of the FAS solution

A = volume of the pulp filtrate used in the oxidation, mL

W = oven-dried weight of pulp (g)

Calculation of γ-cellulose content:

γ-cellulose (%) = [6.85 (V_4-V_3) x N x 20]/[25xW]

where:

V_3 = titration of the solution after precipitation of 🚲-cellulose, mL

V_4 = blank titration, mL

Calculation of β-cellulose:

β-cellulose (%) = 100 - (α-cellulose % + γ-cellulose %)

Table S3. Summary on the composition of crop wastes.

	Raw biomass								
**Content (*wt%*)***	**RH (III)**	**RH (II)**	**RH (I)**	**WS**	**BG (I)**	**BG (II)**	**CS**	**CC**	**Jute**
**Ash**	15.6	17.1	18.6	12.3	2.8	3.3	2.5	1.4	3.4
**Pentosan**	15.8	11.2	12.4	21.4	24.2	21.6	15.4	27.9	23
**Lignin**	24.1	21.7	22.6	17.7	20.4	21.6	26	15.2	14
**Hollocellulose**	60.5	52.5	52.5	63.2	71.1	68.3	64	74	n.d
**α-Cellulose**	37.1	28.5	34.2	36.7	41.2	39.8	36	n.d.	n.d.
**β-Cellulose**	9.8	11.7	6.8	17.1	15.2	15.3	n.d.	n.d.	n.d.
**γ-Cellulose**	13.5	12.2	11.4	9.4	14.6	12.1	n.d.	n.d.	n.d.

*Various content present in crop wastes was determined using TAPPI method. n.d. stands for not done.

5.6. Determination of nutrients present in crop wastes

The nutrients (metal components) present in various crop wastes were determined using ICP-OES analysis. For the ICP-OES analysis, the samples were prepared as follows; O.D. (3 g) raw biomass was taken in a quartz boat was heated in a muffle furnace at 650 °C for 4 h in the presence of air to remove carbon in the form of CO_2. After 4 h the sample (ash) was cooled to RT. Then the silica present in the sample was removed using hydrofluoric acid (HF) treatment because silica can react with HF and form hexafluorosilicic acid (H_2SiF_6) which is miscible with water and can be evaporated. After HF treatment, the solution formed (hexafluorosilicic acid)

was evaporated and the residue left over was dissolved in freshly prepared aqua rgia (HNO_3+3HCl, molar). Then the solution was diluted using Millipore water and was analyzed using ICP-OES technique to quantify the metal nutrients present in various crop wastes.

Table S4. Concentration of various metal nutrients (mmol/g) present in crop wastes.

Nutrients* (mmol/g)	RH (III)	RH (II)	RH (I)	WS	BG (I)	CS	CC
**Na**	0	0	0	0	0	0	0
**K**	0.07	0.07	0.05	0.45	0.03	0.01	0.04
**Ca**	0.03	0.02	0.01	0.06	0.03	0.01	0.01
**Mg**	0.04	0.01	0.04	0.07	0.02	0.01	0.01
**Al**	0.02	0.01	0.01	0.02	0.01	0.04	0.03
**P**	0.05	0.01	0.05	n.d.	0.01	n.d.	n.d.

*Determined with ICP-OES analysis. The calculations were done based on 1 g of crop waste. n.d. stands for not done.

The crop wastes analyzed with ICP-OES method showed that there are various metals present in crop waste in different concentrations (Table S4).

6. Recycling of BAIL for the processing of BG (I) into furfural

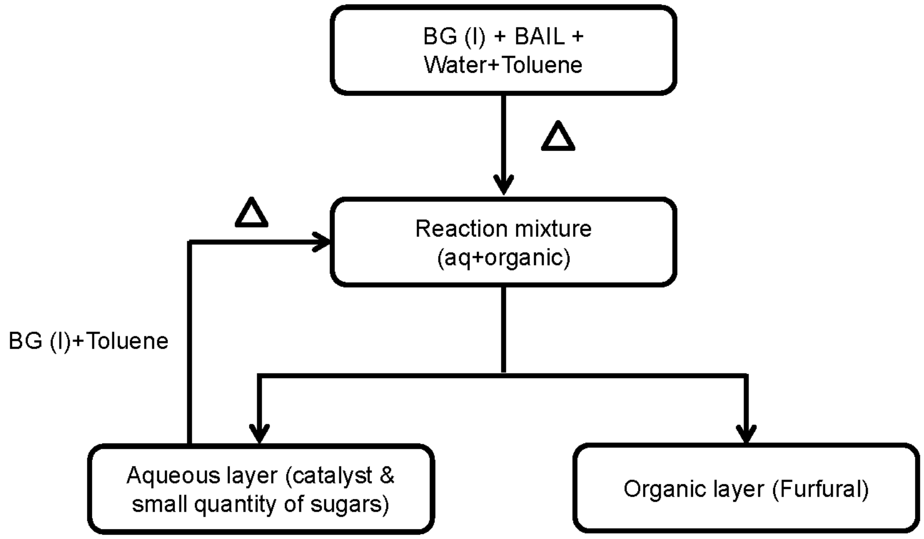

Fig. S6. Process of recycling study for processing of BG (I) into furfural.
